# Supplementary material for: RRM2 Regulates Hepatocellular Carcinoma Progression Through Activation of TGF-β/Smad Signaling and Hepatitis B Virus Transcription
Source: Genes (Basel). 2024 Dec 6;15(12):1575. doi: 10.3390/genes15121575 (PMC11675542; doi:10.3390/genes15121575)
Supplement: Supplementary file 1 [file genes-15-01575-s001.zip › genes-3292678-supplementary.pdf]

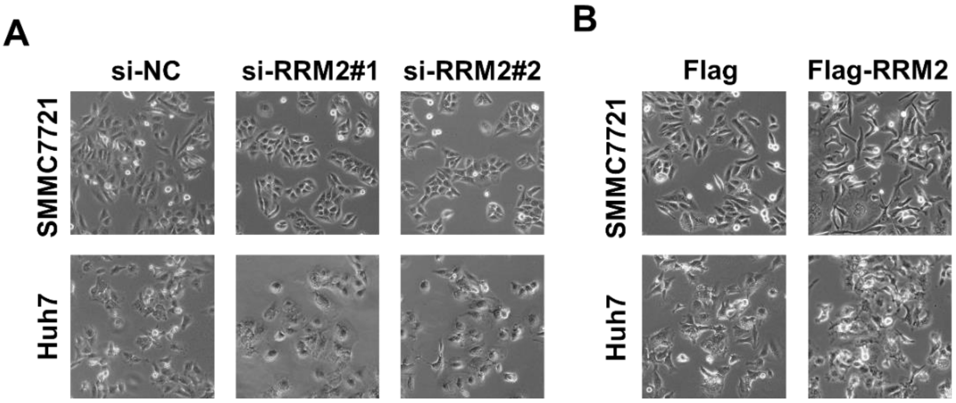

Supplementary Figure S1. RRM2 induces EMT like phenotype. (A-B) Microscopic images of the morphology of SMMC7721 and Huh7 cells with knockdown or overexpression of RRM2.

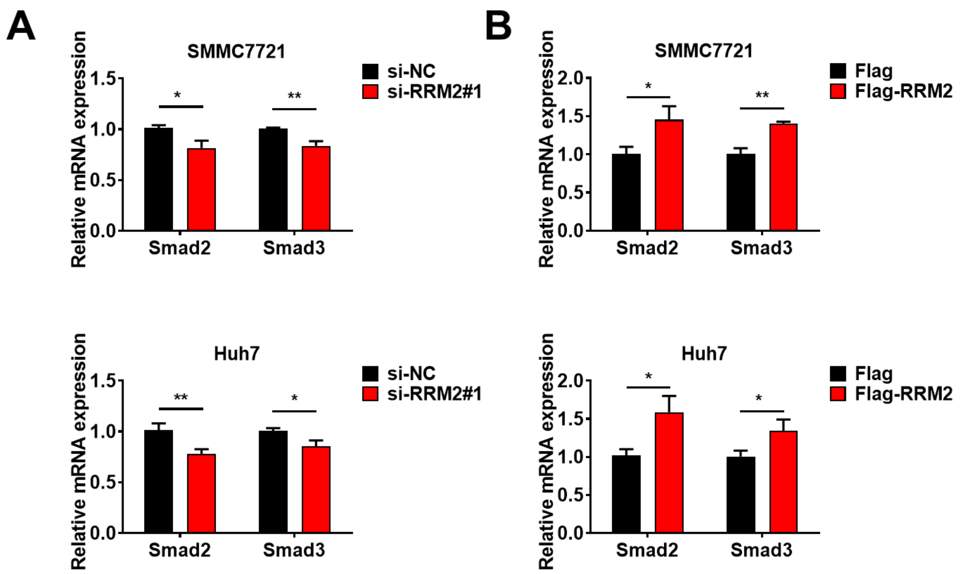

Supplementary Figure S2. RRM2 upregulates the mRNA expression levels of Smad2 and Smad3. (A) Q-PCR assay to detect Smad2 and Smad3 mRNA levels in SMMC7721 and Huh7 cells after RRM2 knockdown. (B) Q-PCR assay to detect Smad2 and Smad3 mRNA levels in SMMC7721 and Huh7 cells after RRM2 overexpression.

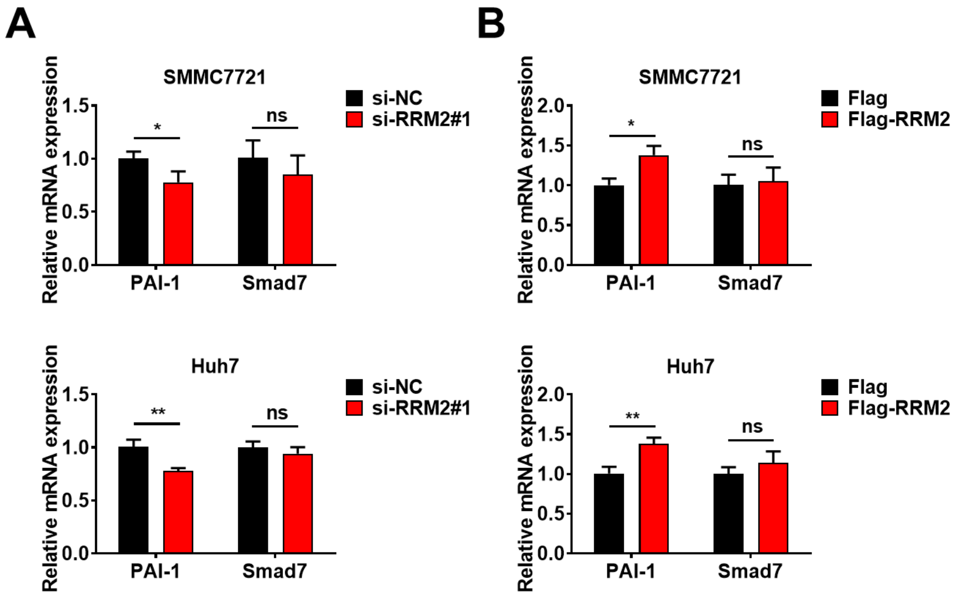

Supplementary Figure S3. Effect of RRM2 knockdown on mRNA expression levels of PAI-1 and Smad7. (A) Q-PCR assay to detect PAI-1 and Smad7 mRNA levels in SMMC7721 and Huh7 cells after RRM2 knockdown. (B) Q-PCR assay to detect PAI-1 and Smad7 mRNA levels in SMMC7721 and Huh7 cells after RRM2 overexpression

Supplementary Table S1: Lists of primers for Q-PCR assay

Genes	Forward Primer	Reverse Primer
cccDNA	GTGCCTTCTCATCTGCCGG	GGAAAGAAGTCAGAAGGCAA
E-cadherin	CCAAAGCCTCAGGTCATAAACA	TCTCCTCCGAAGAAACAGCAAG
HBV DNA	CACCTCTGCCTAATCATC	GGAAAGAAGTCAGAAGGCAA
N-cadherin	CAAGCACCCCTTCACCCAAC	TGGCGAACCGTCCAGTAGG
pgRNA	CTCAATCTCGGGAATCTCAATGT	AGGATAGAACCTAGCAGGCATAAT
Slug	TGATGTGAAGGGTGGAAGATGGA	GAAGAAGCAGGCAAGGAAAAGGT
Snail	GCCTCGCTGCCAATGCT	CGCTGAGGTATTCCTTGTTGC
RRM2	GTGGAGCGATTTAGCCAAGAA	CACAAGGCATCGTTTCAATGG
Vimentin	CCAGATGCGTGAAATGGAAGAG	GTGGGTATCAACCAGAGGGAGT
TGF-β1	GGCCAGATCCTGTCCAAGC	GTGGGTTTCCACCATTAGCAC
GAPDH	GGAGCGAGATCCCTCCAAAAT	GGCTGTTGTCATACTTCTCATGG
Smad2	CGTCCATCTTGCCATTCACG	CTCAAGCTCATCTAATCGTCCTG
Smad3	CCATCTCCTACTACGAGCTGAA	CACTGCTGCATTCCTGTTGAC
Smad7	TTCCTCCGCTGAAACAGGG	CCTCCCAGTATGCCACCAC
PAI-1	GCACCACAGACGCGATCTT	ACCTCTGAAAAGTCCACTTGC
